# Preparation of manganese-doped carbon quantum dots@halloysite nanotube composites films for advanced UV shielding properties

**DOI:** 10.55730/1300-0527.3701

**Published:** 2024-11-20

**Authors:** Xiao ZHANG, Bin WANG

**Affiliations:** School of Chemical and Blasting Engineering, Anhui University of Science and Technology, Huainan, P.R. China

**Keywords:** Halloysite nanotubes, surface modification, ultraviolet shielding, polyvinyl chloride, composite materials

## Abstract

The development of ultraviolet (UV) shielding materials is of great importance to protect human health and prevent the degradation of organic matter. However, the synthesis of highly efficient UV shielding polymer nanocomposites is currently limited by the agglomeration of inorganic anti-UV nanoparticles (NPs) within the polymer matrix and the limited absorption spectrum of UV shielding agents. In this study, highly effective manganese doped carbon quantum dots@halloysite nanotube composites (Mn-CDs@HNTs/PAS) were successfully synthesized by loading manganese-doped carbon quantum dots (Mn-CDs) into UV shielding effective halloysite nanotubes (HNTs) via the solvothermal method, followed by polymerization modification (PAS). The results show that inorganic NPs are evenly dispersed into the polymer matrix. The resulting UV shielding film prepared by mixing Mn-CDs@HNTs/PAS composites with polyvinyl chloride (PVC) shows enhanced UV absorption and thermal stability compared with pure PVC film. It thus appears that Mn-CDs@HNTs/PAS composites have a promising future in UV protection.

## 1. Introduction

Ultraviolet (UV) light plays a vital role in our lives as a significant component of sunlight, constituting approximately 6% of its energy [1, 2]. The wavelength of 100–400 nm in the solar spectrum is collectively called ultraviolet rays. This spectrum can be further divided into UVA (320–400 nm), UVB (280–320 nm), UVC (100–280 nm), and UVD (100–200 nm) based on wavelength [3, 4]. However, UV light is a double-edged sword. While an appropriate level of UV light can enhance vitamin D synthesis in the human body and offer sterilizing and decomposing capabilities in numerous applications, prolonged exposure can lead to sunburn, melanin deposition, accelerated skin aging, and even skin tumors [5]. Additionally, UV can trigger a range of physical and chemical reactions. Prolonged exposure can lead to the aging and decomposition of polymer materials, reducing their performance and causing economic losses [6, 7]. Therefore, it is particularly important to endow polymer materials with good UV shielding. The integration of inorganic nanoparticles (NPs) into the polyvinyl chloride (PVC) matrix is usually an effective strategy to enhance the properties of composite materials [8 – 10].

Halloysite nanotubes (HNTs) naturally occur in aluminosilicate clay minerals and are distinguished by their unique tubular microstructure [11]. They are readily available, inexpensive, easy to process, nontoxic, and biocompatible [12, 13]. Their one-dimensional structure, high aspect ratio, and active groups on both internal and external surfaces facilitate the modification of composite materials, providing versatile properties. This enables them to have wide-ranging prospects in polymer materials. Unlike other NPs [14], HNTs can be easily dispersed into the polymer matrix due to their reduced intramolecular contact between NPs and enhanced intermolecular contact with polymers. Importantly, self-agglomeration of HNTs is limited by the minor hydrogen bonding force between them, thereby maintaining a better dispersion state in a variety of polymer matrices and solutions.

Wang et al. modified HNTs with silver (Ag) and photoresponsive α-Fe_2_O_3_ NPs to prepare Ag-Fe_2_O_3_/HNT complexes [15]. Xu et al. grew ZnO nanoparticles in situ on the surface of HNTs to produce superamphiphobic fabrics with UV shielding and antistatic properties [16]. Peng et al. assembled ZnO nanoparticles on the surface of HNTs to simultaneously exhibit homogeneous dispersion, excellent absorptivity, and superior photocatalytic activity [17]. However, to the best of our knowledge, it has never been reported that manganese-doped carbon quantum dot (Mn-CD) loaded HNTs can improve the UV shielding rate of composite films. CDs are a new type of quasizero-dimensional carbon nanomaterials famous for their cold luminescence properties. Their unique chemical properties as ultraviolet shielding agents have attracted great interest [18 – 20]. In the present study, the prepared Mn-CDs were loaded onto HNTs to prepare composite nanomaterials with UV shielding performance. The PVC composite film prepared by the nanoparticles has excellent UV shielding performance and can be evenly dispersed in the PVC matrix.

## 2. Materials and methods

### 2.1 Materials

*N,N*-dimethylformamide (DMF) was purchased from Sinopharm Group Chemical Reagent Co. Ltd. PVC powder (SG5, average polymerization degree of about 1000) was provided by Yousuo Chemical (Shandong) Technology Co. Ltd. Other reagents including manganese acetate tetrahydrate, o-phenylenediamine (OPD), halloysite nanotubes (HNTs), 4,4′-diaminodiphenyl sulfone (DDS), dianhydride triphenyltetraformic acid (PMDA), and Rhodamine B (Rh B) were purchased from Aladin Reagents (Shanghai) Co. Ltd. All chemicals were of analytical grade and were used directly without any further purification. The water used in the experiment was resin deionized purified water.

### 2.2 Preparation of Mn-CDs@HNTs

First 0.61 g of manganese tetrahydrate acetate and 0.11 g of OPD were added to 30 mL of absolute ethanol and they were stirred. Next, 0.96 g of HNTs was added and mixed evenly and then the solution was mixed in an ultrasonic instrument for 30 min. The resulting solution was poured into a 100-mL Teflon lined reactor, kept at 180 °C for 12 h, and then cooled to room temperature [21]. The resulting suspension from the reaction was centrifuged at 10,000 rpm to obtain the precipitate and washed several times with deionized water and absolute ethanol. Finally, the obtained product was dried at 70 °C for 12 h.

### 2.3 Preparation of Mn-CDs@HNTs/PAS

Firstly, 0.77 g of PMDA was weighed and completely dissolved in 15 mL of DMF and sonicated for 30 min, denoted as solution A. Next, 0.51 g of Mn-CDs@HNTs and 0.81 g of DDS were weighed and completely dissolved in 15 mL of DMF and sonicated for 30 min, denoted as solution B. Then, under constant stirring, solution A was added to solution B in batches within 1 h with an interval of 10 min. After being completely dissolved, the reaction was completed by continuing magnetic stirring for 2 h [22, 23]. Finally, the resulting suspension was centrifuged at 10,000 rpm to obtain the precipitate, which was washed several times with deionized water and absolute ethanol. Finally, the obtained product was dried at 70 °C for 12 h.

### 2.4 Preparation of PVC composite film

Firstly, 0.4 g of PVC resin was weighed and completely dissolved in 8 mL of DMF under stirring and denoted as solution A. The specified amount of UV absorber was weighed and dispersed in 2 mL of DMF solution for 30 min, which was denoted as solution B. Solution B was added to solution A dropwise and stirred for 12 h under magnetic stirring. The obtained uniform dispersion solution was poured into a petri dish, placed in the oven, dried at 70 °C for 12 h, and then the petri dish was immersed in water for a period of time to obtain PVC composite film. Pure PVC films were prepared by the same method for comparison of UV absorption properties.

The UV absorber content was 0.3 wt%, 0.6 wt%, 0.9 wt%, and 1.2 wt% of Mn-CDs@HNTs/PAS and 0.9 wt% of Mn-CDs@HNTs relative to the quality of PVC resin.

### 2.5 Photocatalytic experiment

The UV shielding ability of PVC composite films can generally be characterized by recording the photodegradation behavior of Rh B solution. To evaluate this, the absorbance of the Rh B solution is measured at different time intervals under exposure to UV light. The absorbance of Rh B solution exposed to UV light at 0 min was recorded as A_0_, and the absorbance measured by removing Rh B samples at every 40 min interval was recorded as A_t_. The degradation rate was calculated by the following formula:


$$I=\frac{A_{t}}{A_{0}}\times 100\%$$

Firstly, the Rh B solution (concentration of 10^−3^ M) was prepared. Rh B (0.01 g) was dissolved in 50 mL of water and 12.5 mL of the solution was placed in a 250-mL brown volumetric flask at constant volume, and it was stored in dark conditions for later use. During the photocatalytic experiment, 50 mL of Rh B solution was placed in a 100-mL beaker and 0.051 g of P_25_ (TiO_2_) catalyst was weighed and poured into the beaker. The solution was placed on a magnetic stirrer for 30 min in dark conditions to mix evenly. After the adsorption equilibrium was reached, 6.0 mL of the solution was transferred into two centrifuge tubes, and the solution was centrifuged to remove the P_25_ solid. The supernatant was placed in a cuvette for absorbance detection.

The PVC composite film was completely covered on the beaker mouth, and the UV lamp was used to illuminate the beaker directly above the beaker. The distance between the UV lamp and the liquid surface was about 15 cm. After irradiation for 0, 40, 80, 120, 160, 200, and 240 min, 6 mL of the solution was withdrawn from the beaker and transferred into a centrifuge tube to remove the TiO_2_ by centrifugation. Following centrifugation, the supernatant was collected in a cuvette for absorbance measurement. After the test, the solution was evenly dispersed and poured back into the beaker for the next period of irradiation. The above steps were repeated for each measurement.

### 2.6 Characterization

FTIR spectra were measured using a Fourier transform infrared spectrometer (Nicolet is50, Thermo Fisher Scientific (USA) Co. Ltd.) to analyze different functional groups on the sample surface. A UV-visible near-infrared spectrophotometer (UV-3600 PLUS, Shandong Youjia Scientific Instrument (Shandong) Co. Ltd.) was used to record the UV spectrum of the product and the UV diffuse absorption spectrum of the PVC composite film. The crystalline phases of the samples were analyzed by X-ray diffraction using an X-ray electron diffractometer (ESCALAB Xi, Thermo Scientific (USA), Co. Ltd.). TEM images were obtained and mapping analysis was done using a transmission electron microscope (Tecnai G2 F20 S-Twin, Thermo Fisher (USA) Co. Ltd). Thermal stability was studied using a thermogravimetric analyzer (TGA/DSC 3, Mettler Towler Technology (China) Co. Ltd. Group) at a heating rate of 10 °C /min in the temperature range from room temperature to 600 °C. In the photocatalytic experiment, a UV–vis spectrophotometer (UV-6000, Shanghai Precision Instrument Co. Ltd.) was used to record the photodegradation rate of the UV–vis absorption spectrum of the sample by UVA 340 (15 W) UV lamp.

## 3. Results and discussion

### 3.1 FTIR and XRD analysis

**Figure 1:**
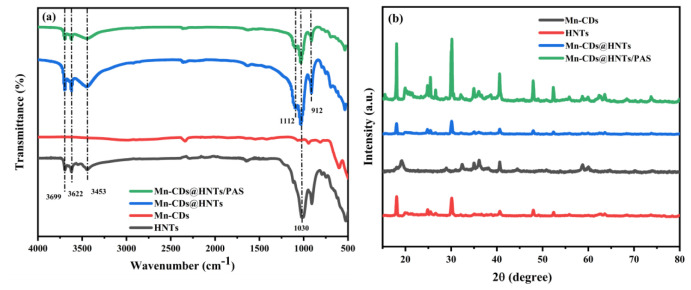
(a) FTIR spectra, (b) X-ray diffractogram.

The vibrational bands and interfacial interactions of the Mn-CD-loaded HNTs were characterized by FTIR. As shown in Figure 1a, the Mn-CDs loaded on HNTs exhibit the same specific diffraction peaks as the HNTs [24], confirming the successful binding of the HNTs to the quantum dots [25]. In the high-frequency region, HNTs show a strong split absorption peak at 3699 cm^−1^, which is attributed to the Al–OH group stretching vibration of HNTs and the Si–OH group hydroxyl stretching vibration between the aluminum–oxygen octahedron and the silicon–oxygen tetrahedron. The characteristic absorption peak of the hydroxyl group of HNTs is at 3622 cm^−1^. The broad peak at 3453 cm^−1^ is due to the −Si–OH-bending vibration. In the low frequency region, 1112 and 1030 cm^−1^ are the stretching vibration peaks of the −Si–O– bond [26], and the absorption peak of 912 cm^−1^ is attributed to the bending vibration of Al–O–H in the Al–(OH•O) octahedron.

Moreover, the addition of Mn-CDs not only enhances the absorption peak at 3622 cm^−1^, but also gradually expands the absorption peak in the range of 3300–3700 cm^−1^, indicating that the addition of Mn-CDs enhances the vibration of −NH_2_ and −OH. After the PAS polymerization modification of the product, the addition of PMDA and DDS may increase the amount of −OH on the surface of Mn-CDs@HNTs, increase the H removed after the participation of −OH in the reaction, and increase the O–H stretching vibration intensity. The change in the peak of the state showing high absorption intensity proves the interaction of Mn-CDs and HNTs, confirming that Mn-CDs are loaded and modified successfully [27]. At the same time, the existence of these functional groups enhances the intermolecular force and improves the compatibility of NPs and PVC in physical blending, which effectively solves the problem of uneven dispersion of NPs in the PVC matrix.

The crystallization states of HNTs and each synthetic product were analyzed by XRD patterns. Figure 1b shows that the composites Mn-CDs@HNTs and Mn-CDs@HNTs/PAS exhibit diffraction peaks at the same position as HNTs, which are 2θ = 12.11°, 20.07°, 24.56°, 35.01°, 54.52°, and 62.56°, respectively, corresponding to HNTs1: Type 1 layered silicate (001) crystal plane [28–30]. In addition, the XRD patterns of Mn-CDs@HNTs and Mn-CDs@HNTs/PAS after Mn-CDs composite show that the diffraction peaks at 2θ = 40.63° and 58.78° are the same as those of Mn-CDs. After PAS modification of Mn-CDs@HNTs, the diffraction peak is obviously sharp, indicating that, after PAS modification, Mn-CDs and HNTs composite has a good crystallite structure. Therefore, Mn-CDs@HNTs and Mn-CDs@HNTs/PAS were successfully synthesized through the hydrothermal process, and the crystalline state of the HNTs was not affected.

### 3.2 TEM analysis

**Figure 2:**
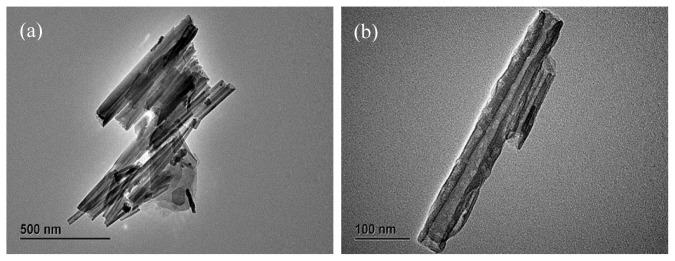
TEM image of Mn-CDs@HNTs/PAS.

Figure 2 presents a TEM image captured by a projection electron microscope, displaying key parameters such as the microscopic morphology, roughness, structural details, and surface size of the Mn-CDs@HNTs/PAS sample. Figure 2a further confirms the successful synthesis of Mn-CDs and HNTs with good crystallinities. Figure 2b shows that HNTs are medium controlled tubes with an average length of 200–400 nm and an inner diameter of 20–30 nm. HNTs have an amorphous surface rich in surface hydroxyl groups, which are potential reaction sites for surface modification. Moreover, surface edges and defects can provide higher specific surface energy and serve as reaction sites for metal deposition on the outer and inner surfaces [31–32]. The TEM images demonstrate that after PAS polymerization modification, the nanotubes retain their rod-like morphology and the stability of their structure remains unchanged.

**Figure 3:**
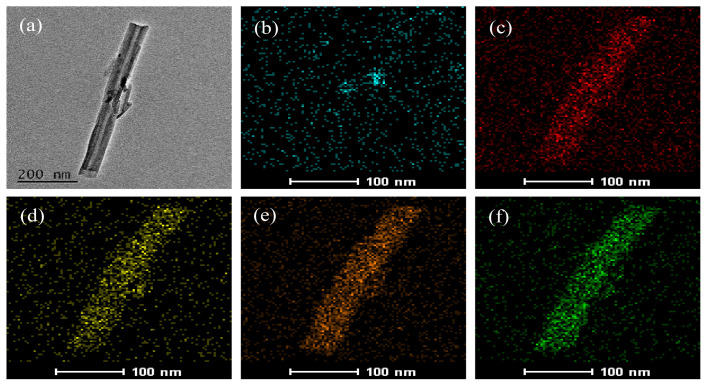
TEM microphotographs and chemical maps of Mn-CDs@HNTs/PAS (a) and elemental maps of Mn (b), N (c), Al (d), O (e), and Si (f).

In Figure 3, the morphology of Mn-CDs@HNTs/PAS nanocomposite and its nanostructure characteristics were studied by field emission scanning electron microscopy to confirm the mixing nature of Mn-CDs and nanotubes. Figure 3a shows a rod-like micrograph of the synthesized Mn-CDs@HNTs/PAS sample, revealing nanotubes with lengths between 300 and 400 nm. Figures 3b–3f show the TEM map with uniform distribution of Mn, N, Al, O, and Si elements, where Mn and N are derived from quantum dots [ 33, 34 ], indicating that Mn and N elements are successfully doped into HNTs.

### 3.3 UV–vis analysis

**Figure 4:**
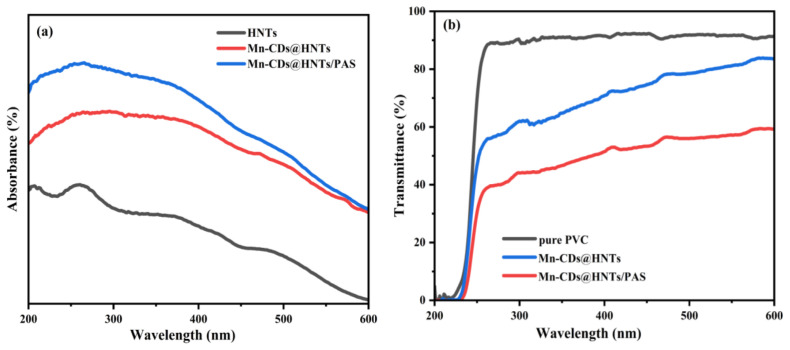
UV–vis spectra: (a) UV absorption of NPs, (b) UV transmittance of different PVC composite films with 0.9 wt% content.

The UV spectra of the HNTs and their complexes are shown in Figure 4a. It is evident that from the UV to visible light range, the absorption spectrum of the material is significantly enhanced after compositing with Mn-CDs, especially in the UVA and UVB regions. This indicates that the incorporation of Mn-CDs improves the UV absorption ability of HNTs. In addition, the UV absorption value of the material Mn-CDs@HNTs/PAS after the organic modification of PMDA and DDS is higher than that of the unmodified product, indicating that the benzene ring and amino group contribute to enhance the photon capture of the composite product.

Figure 4b shows the UV transmittance of pure PVC and composite films with the same content of different NPs in the range of 200 to 600 nm. The results further demonstrate that Mn-CDs@HNTs/PAS enhances UV absorption, and the Mn-CDs@HNTs/PAS/PVC composite exhibits the most effective UV shielding performance.

The optical properties of the prepared materials were also analyzed using the curves of (αhv)^2^ vs photo energy (hv). The optical energy gap (Eg) can be deduced according to Tauc’s expression (1) [ 35]:


(1) $${\left(\alpha hv\right)}^{1/n}=A(hv-Eg),$$

where α is the absorption coefficient, *hν* is the photon energy, A is the absorption index, and n represents the type of electron transition, usually between 1/2 and 3.

**Figure 5:**
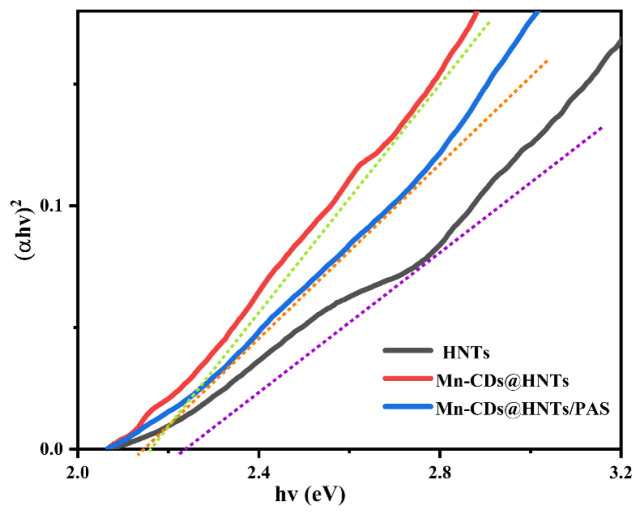
The inherent Tauc’s plot of NPs.

As shown in Figure 5, the intersection of the tangent line of the curve in the (*αhν*)^2^ - Eg diagram and the horizontal axis is the band gap energy of the optical catalyst. From the figure, it can be concluded that the energy band gap of the HNTs is 2.24 and, when Mn-CDs are compounded, the capacity band gap of the composite is reduced to 2.15. The smaller the gap width of Mn-CDs@HNTs, the stronger the optical absorption range and intensity. The change in the optical band gap Eg indicates a change in the optical band structure of Mn-CDs@HNTs. This narrow band gap can improve the efficiency of UV light collection, thus improving the UV light shielding performance [ 36].

**Figure 6:**
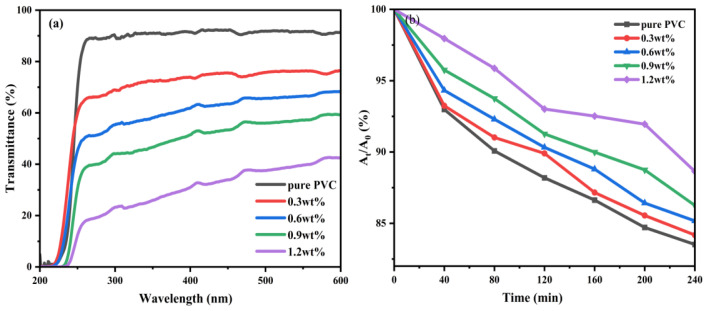
(a) UV transmittance of PVC composite films with different contents, (b) degradation diagram of Rh B solution by PVC composite films with different contents.

Figure 6a presents the UV transmittance of pure PVC film and Mn-CDs@HNTs/PAS/PVC composite films with varying Mn-CDs@HNTs/PAS content. It is evident that PVC composite films with different Mn-CDs@HNTs/PAS concentrations exhibit varying degrees of UV shielding, while the UV transmittance of the pure PVC film reaches as high as 90%. As the Mn-CDs@HNTs/PAS content increases, the transmittance of the PVC composite films gradually decreases, indicating that the nanoparticles are uniformly dispersed within the PVC matrix.

### 3.4 UV shielding properties of pure PVC films and PVC composite films

The UV absorption properties of nanocomposite films were studied by UV–vis absorption spectroscopy. The transmittance data were used to calculate the ultraviolet protection factor (UPF) using the following equation:


(2) $$UV\;protection\;factor\;(UPF)=\frac{\int_{280}^{400}E(\lambda )S(\lambda )d\lambda }{\int_{280}^{400}E(\lambda )S(\lambda )T(\lambda )d\lambda }$$

where E (λ) is the relative erythema action spectrum, S (λ) is the spectral irradiance (Wm^−2^ nm^−1^), T (λ) is average spectral transmittance of fabric, dλ is bandwidth, and λ is wavelength.

The blocking rate is calculated by Eq. (3):


(3) $$UV\;shielding\;ability\;(\%)=\left(1-\frac{\int_{a}^{b}T(\lambda )d(\lambda )}{\int_{a}^{b}d(\lambda )}\right)100\%$$

where T(λ), d(λ), and λ are the average spectral transmittance of the film, bandwidth, and wavelength, respectively. The values of a and b were 320 and 400 nm for UVA, 280 and 320 nm for UVB, and 200 and 280 nm for UVC, respectively. Table shows the percentage of UVA and UVB radiation blocking and UPF of pure PVC and PVC nanocomposite films. Pure PVC has a very low UPF value of 1.10 and can only block 9.0% of UVA and 10.5% of UVB. When only 0.3% NPs is added, the blocking rate of UVA and UVB can reach 27.5% and 31.4%, respectively. It can shield up to 73% UVA and 77.7% UVB, and the UPF value is also increased.

The UV shielding ability of pure PVC and its composite film was further tested by photocatalytic degradation of Rh B solution. As shown in Figure 6b, for the Mn-CDs@HNTs/PVC film (0%, 0.3%, 0.6%, 0.9%, 1.2%), it is obvious that the degradation rate of Rh B solution of the composite film after adding Mn-CDs@HNTs/PAS is significantly weakened by UV light irradiation. At 240 min of irradiation, the degradation rates (A_t_/A_0_) % of Rh B solution were 83.5, 84.2, 85.2, 86.3, and 88.6, respectively, and the effect is better than the same content of nano-ZnO composite film, indicating that the prepared Mn-CDs@HNTs/PAS/PVC composite film had excellent UV shielding performance [37].

### 3.5 Thermal stability of films

**Figure 7:**
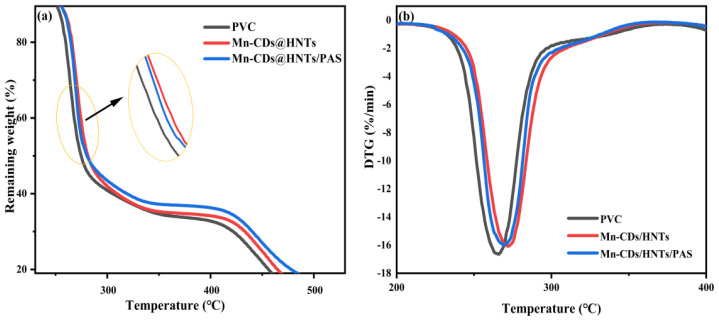
Pure PVC and NPs composite film: (a) TG curves, (b) DTG curves.

The thermal stability of PVC is an important factor restricting its smelting process and it is very important in real life. The thermogravimetric analysis (TGA) and derivative thermogravimetric (DTG) scans of pure PVC and composite films with the same content of Mn-CDs@HNTs/PVC and Mn-CDs@HNTs/PAS/PVC are presented in Figures 7a and 7b.

As shown, the Mn-CDs@HNTs/PAS/PVC composite film had a relatively high residual weight from room temperature to 600 °C in Figure 7a, indicating that Mn-CDs@HNTs/PAS improves the thermal stability of pure PVC film. It can be seen from the figure that there is a slow weight loss process below 100 °C mainly caused by the evaporation of residual solvent and moisture [38]. Between 200 and 300 °C, the composite film completely lost about 45.19% of its weight due to the decomposition of PVC. After heating the PVC composite film to 400 °C, due to the decomposition of residual groups, the final residual weight was about 11.0%, 14.3%, and 17.6%, respectively. The TGA curves of Mn-CDs@HNTs/PVC composite film and Mn-CDs@HNTs/PAS/PVC composite film overlapped between 200 °C and 300 °C, and the weight loss rate was lower than that of pure PVC, indicating that the addition of Mn-CDs could enhance thermal stability. After 300 °C, the thermal stability of the modified Mn-CDs@HNTs/PVC composite film was always higher than that before modification, indicating that the decomposition rate of residual groups is reduced and the thermal stability of the film is enhanced by PAS polymerization. Moreover, the DTG curve shows (Figure 7b) that the weight loss rate of pure PVC film reaches its maximum at about 265 °C, while the weight loss rate of composite film with only 0.9 wt% nanoparticle content reaches its maximum at 274 °C, which is significantly later than that of pure PVC [39]. The results show that Mn-CDs@HNTs/PAS NPs can enhance the thermal stability of PVC films.

## 4. Conclusion

In summary, Mn-CDs@HNTs/PAS composite was successfully prepared using the solvothermal method followed by chemical precipitation, showing dispersibility and uniform size distribution as well as excellent UV resistance. As a multifunctional additive, Mn-CDs@HNTs/PAS was evenly integrated into PVC films via solvent evaporation. The experimental results show that the introduction of Mn-CDs@HNTs/PAS endows PVC films with improved dispersion as well as thermal and UV shielding performance. Therefore, the developed Mn-CDs@HNTs/PAS composite provides extensive potential for use across a variety of polymer materials.

## Figures and Tables

**Table:** Percentage of blocking from UV-A and UV-B and UPF values of samples.

Samples	Percentage (%)		Blocking

	UVA	UVB	UPF value
Pure PVC	9.01	10.48	1.10
0.3wt%	27.48	31.37	1.40
0.6wt%	41.13	45.36	1.74
0.9wt%	53.32	56.80	2.16
1.2wt%	73.00	77.71	3.93
